# Role of α-adrenergic activation during the hemodynamic response to maternal hyperoxygenation in fetal sheep^†^

**DOI:** 10.1093/biolre/ioag165

**Published:** 2026-08-04

**Authors:** Jordan A Minns, Jack R T Darby, Steven K S Cho, Brahmdeep S Saini, Stacey L Holman, Christopher K Macgowan, Mike Seed, Janna L Morrison

**Affiliations:** Early Origins of Adult Health Research Group, Health and Biomedical Innovation, School of Pharmacy and Biomedical Sciences, Robinson Research Institute, Adelaide University, Adelaide, Australia; Early Origins of Adult Health Research Group, Health and Biomedical Innovation, School of Pharmacy and Biomedical Sciences, Robinson Research Institute, Adelaide University, Adelaide, Australia; Translational Medicine, Hospital for Sick Children, Toronto, Canada; Division of Cardiology, Hospital for Sick Children, Toronto, Canada; Department of Pediatrics, University of Toronto, Toronto, Canada; Translational Medicine, Hospital for Sick Children, Toronto, Canada; Division of Cardiology, Hospital for Sick Children, Toronto, Canada; Early Origins of Adult Health Research Group, Health and Biomedical Innovation, School of Pharmacy and Biomedical Sciences, Robinson Research Institute, Adelaide University, Adelaide, Australia; Translational Medicine, Hospital for Sick Children, Toronto, Canada; Department of Medical Biophysics, University of Toronto, Toronto, Canada; Translational Medicine, Hospital for Sick Children, Toronto, Canada; Division of Cardiology, Hospital for Sick Children, Toronto, Canada; Department of Pediatrics, University of Toronto, Toronto, Canada; Early Origins of Adult Health Research Group, Health and Biomedical Innovation, School of Pharmacy and Biomedical Sciences, Robinson Research Institute, Adelaide University, Adelaide, Australia; Department of Physiology, Faculty of Medicine, University of Toronto, Toronto, Canada

**Keywords:** fetal hemodynamics, oxygenation, MRI, maternal hyperoxygenation and streaming

## Abstract

Normal neurodevelopment relies on effective placental oxygen transfer and the specialized circulatory shunts that direct oxygen-rich blood to key fetal organs. The tone of these shunts, particularly the ductus venosus, is critical for routing oxygen and nutrient-rich blood towards the fetal brain. However, these shunts are sensitive to both oxygenation status and sympathetic innervation. Notably, fetal hypoxia increases shunting through the ductus venosus via enhanced hepatic sympathetic activation, a mechanism that redistributes oxygen rich blood toward vital organs. Thus, we hypothesized that hyperoxygenation may decrease shunting through the ductus venosus via hepatic sympathetic regulation but may be reversed if combined with an α-adrenergic agonist. Pregnant ewes (*n* = 8) underwent fetal catheterization surgery at 116–117 days gestation. At 120–124 days gestation, ewes were anaesthetized, and magnetic resonance imaging (MRI) scans were performed during normoxia, hyperoxia, and hyperoxia + phenylephrine infusion to measure blood flow and oxygenation within the major fetal vessels using phase-contrast and T2 oximetry MRI techniques, respectively. Hyperoxia had minimal effects on fetal hemodynamics. However, with the addition of phenylephrine, there was increased flow through the ductus venosus and ascending aorta but decreased flow toward the brain via the carotid arteries. Hyperoxia + phenylephrine increased overall fetal oxygen delivery but decreased cerebral oxygen delivery, despite no change in ductus venosus shunting (ductus venosus/umbilical vein flow ratio). There was no change in fetal or cerebral oxygen consumption. In conclusion, hyperoxia + phenylephrine reduced cerebral oxygenation, indicating that increased ductus venosus flow does not translate to improved cerebral oxygen delivery when carotid vascular resistance is elevated.

## Graphical Abstract

**Graphical Abstract:**
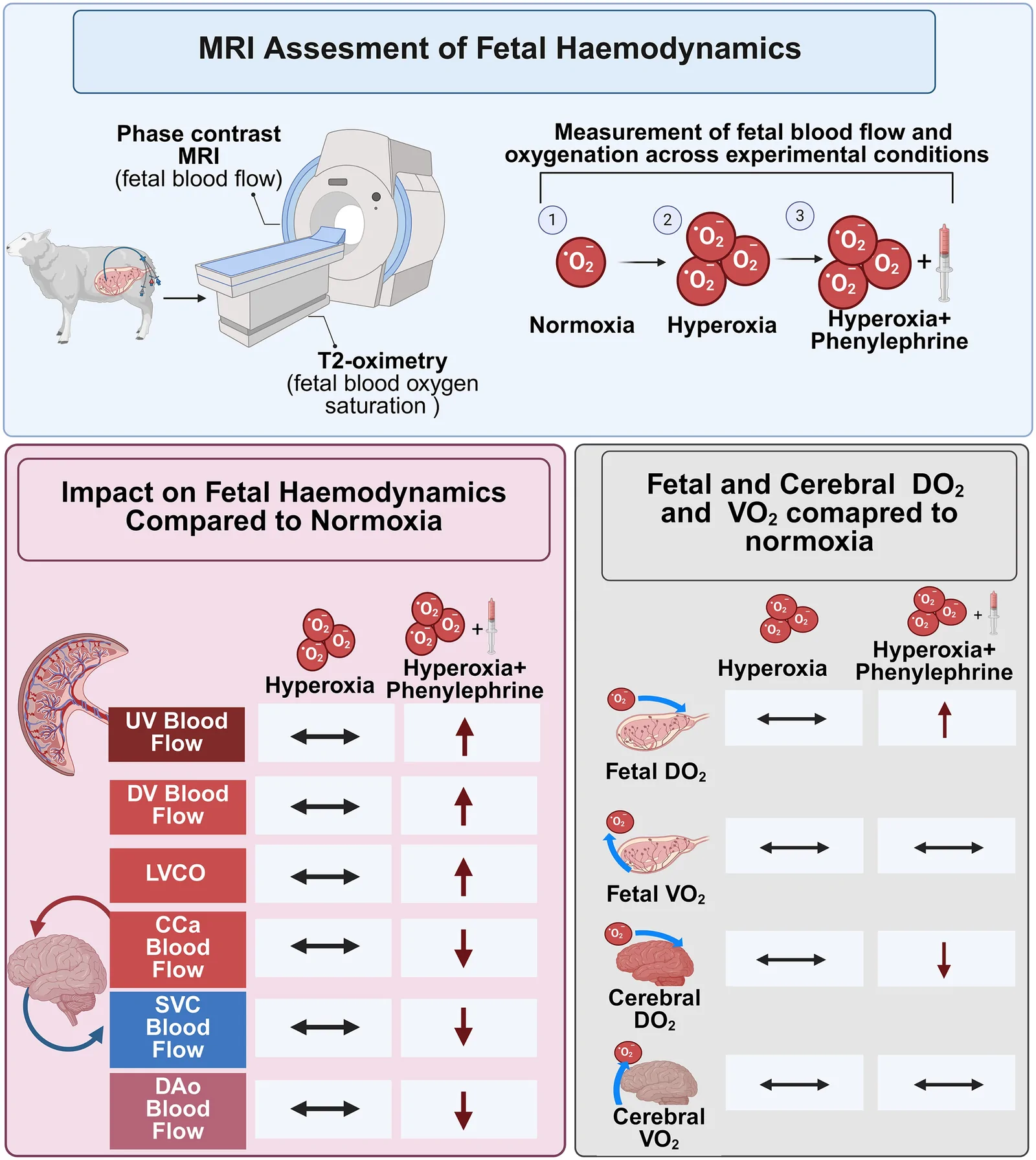

## Teaser

Hyperoxia + phenylephrine increased ductus venosus and aortic flow but reduced carotid flow, ultimately reducing cerebral oxygen delivery.

## Introduction

Despite the relatively low oxygen environment *in utero,* the fetal circulatory system is uniquely adapted to ensure optimal oxygen delivery to key organs such as the brain [1–3]. Unlike adult life when the majority of oxygen exchange occurs in the lungs, the fetus receives oxygen and nutrients from the maternal circulation through the placenta [4, 5]. Oxygenated blood returning from the placenta is carried by the umbilical vein (UV) towards the fetus, where it can either flow through the hepatic vasculature, becoming deoxygenated, or flow directly into the inferior vena cava (IVC) through a specialized shunt called the ductus venosus (DV) [2, 6, 7]. Typically, a substantial fraction of UV flow bypasses the liver via the DV [3, 8] and the blood flow spirals to the posterior portion of the IVC toward the left side of the heart via the foramen ovale, and ultimately to the brain [3, 6]. Importantly, the amount of oxygen and nutrient-rich blood that bypasses the hepatic circulation is determined by the tone of the DV.

The tone of the DV refers to the degree of constriction or relaxation in the vessel wall and responds to physiological conditions such as fetal hypoxemia [8–11]. The fetal response to hypoxemia is a rise in circulating catecholamines and other vasoconstrictors, thereby increasing hepatic vascular resistance and diverting UV blood flow through the DV and toward the brain [12, 13]. Notably α-adrenergic blockade blunts this response [2, 9, 11, 13, 14]. This mechanism allows for more streaming of oxygen rich blood towards the fetal brain without first perfusing the hepatic vasculature. Thus, the proportion of blood flowing through the DV as opposed to the liver is dependent on the oxygen concentration of umbilical vein blood [8, 15].

In cases of fetal distress, maternal hyperoxygenation has been proposed as a therapeutic intervention to improve oxygen delivery. This has been explored in growth restricted fetuses with abnormal Doppler indices [16], and acute intrapartum distress characterized by abnormal fetal heart rate patterns during labor [17]. While maternal hyperoxygenation increases blood flow velocity through the DV in healthy fetuses [18], the physiological cause of this increase remains uncertain. As velocity is influenced by both volumetric flow and vessel diameter, it is unclear whether this rise reflects an increase in oxygen delivery (DO_2_) or increased vascular tone of the DV that narrows the shunt. Thus, the direct impact of hyperoxygenation on DV vascular tone remains poorly defined. Specifically, while it is established that fetal hypoxemia decreases DV tone via α-adrenergic mechanisms to facilitate shunting [13], it is not yet clear if fetal hyperoxia actively reverses this tone or if the observed changes in flow are a passive result of altered arterial pressure.

Blood flow through the DV has previously been studied using radioactively labelled microspheres and ultrasound Doppler velocimetry [8]. However, microspheres are invasive and cannot be used in humans, while ultrasound can visualize the DV, it cannot accurately quantify blood flow, as Doppler techniques measure mean velocity to estimate volumetric flow [19]. Magnetic resonance imaging (MRI) addresses these limitations by offering a non-invasive method to robustly quantify volumetric blood flow. We have previously validated two MRI modalities to measure both blood flow and oxygenation within the fetal circulation [20–22]. Two-dimensional phase contrast (PC) cine MRI enables measurement of both quantitative flow velocities and flow volumes within the major fetal vessels, while T2 oximetry utilizes the magnetic properties of deoxyhemoglobin and oxyhemoglobin to determine oxygen saturation within these vessels [20]. Combining these two MRI modalities allows for not only a comprehensive understanding of fetal hemodynamics but also an effective assessment of oxygen delivery (DO_2_) within the fetal circulation [23].

Herein, we aimed to use a combination of PC and T_2_ Oximetry MRI to investigate the impact of maternal hyperoxygenation with and without an α-adrenergic agonist on fetal blood flow through the DV and the overall impact on cerebral DO_2_. We hypothesized that fetal hyperoxia would result in a decrease in blood flow through the DV and thus result in decreased cerebral DO_2_. We further hypothesized that an α-adrenergic agonist administered during fetal hyperoxia would increase absolute DV flow while also increasing proportional DV shunting, ultimately increasing cerebral DO₂.

## Materials and methods

### Ethics

All experimental protocols were reviewed and approved by the Animal Ethics Committee of the South Australian Health and Medical Research Institute (SAM-389.19) and abided by the Australian Code of Practice for the Care and Use of Animals for Scientific Purposes developed by the National Health and Medical Research Council. The experiment was designed in line with the Animal Research: Reporting of in-vivo Experiments (ARRIVE) guidelines [24, 25]. All investigators were aware of ethical principles [26, 27] and the principles of the 3Rs which include replacing animals with alternative methods, reducing the number of animals used and refinement to minimize any discomfort or distress [28]. Merino ewes were sourced from the SAHMRI Farm (Burra, South Australia, Australia) and housed indoors under controlled environmental conditions (20°C–22°C; 12-h light/dark cycle). Ewes were maintained in individual pens with visual contact of other ewes and provided a diet of lucerne chaff (1 kg) and Ewe & Lamb nuts (300 g) comprised of cereal hay, lucerne hay, barley, oats, almond shells, lupins, oat bran, lime, and molasses (Johnsons & Sons, Kapunda, South Australia, Australia), with *ad libitum* access to water.

### Fetal catheterization surgery

Ewes (*n* = 8) were fasted for at least 12 hours prior to the induction of general anesthesia. At 116–117 days gestational age, ewes bearing singleton fetuses (*n* = 8; three males, five females) were anesthetized with intravenous diazepam (0.3 mg kg − 1) and ketamine (5 mg kg − 1) and maintained with isoflurane (1.5%–2.5% in 100% oxygen). Ewes then underwent aseptic fetal catheterization surgery following previously established protocols [29]. Fluid filled heparinized saline vascular catheters were implanted into the maternal jugular vein, fetal femoral artery and vein, umbilical artery and vein, and the amniotic cavity as previously described [7, 30, 31]*.* As each fetus was exteriorized from the uterus, it received an intramuscular injection of antibiotics consisting of 1 ml of Duplocillin (150 mg/ml procaine penicillin and 112.5 mg/ml benzathine penicillin; Norbrook Laboratories Ltd, Gisborne, Australia)) and 1 ml of 125 mg ml − 1 dihydrostreptomycin (Sigma-Aldrich, St Louis, MO, USA). Following catheterization, the fetus was then returned to the uterus, which was sutured closed, and the catheters were exteriorized through an incision in the ewes’ flank. Antibiotics, 3.5 ml of Duplocillin and 2 ml of 125 mg/ml dihydrostreptomycin were administered intramuscularly to the ewe at surgery and for 3 days post-operatively. All ewes received an analgesic (meloxicam, 0.5 mg/kg, subcutaneously) on the day before and day of surgery [32]. Post-surgery, each fetus received antibiotics for 4 days intra-amniotically (500 mg ampicillin, Lyppards, South Australia, Australia).

### Blood sampling and fetal blood gas measurements

Blood samples from the fetal femoral artery were collected daily to monitor fetal health through the measurement of fetal blood gases including arterial partial pressure of oxygen (PaO_2_), partial pressure of carbon dioxide (PaCO_2_), oxygen saturation (SaO_2_), pH, hemoglobin concentration [Hb], hematocrit (Hct), base and lactate, with a RAPIDPOINT 500 (Siemens Healthineers, Victoria, Australia) temperature corrected to 39°C for sheep blood. During the MRI protocol, fetal femoral artery blood samples were prior to commencement of each scan state.

### Fetal sheep MRI protocol

At 123-124d GA, ewes (*n* = 8) were fasted for at least 12 hours before the induction of general anesthesia as described for surgery above. Ewes were mechanically ventilated at a rate of 15–20 breaths min^−1^ as previously described [7, 33]. However, before maternal anesthesia was induced, a fetal blood sample from the fetal femoral artery was taken to record the normal, unanesthetized fetal blood gas status (RAPIDPoint 500), so that maternal-inspired oxygen could be titrated to match conscious fetal physiology for baseline measurements. For the duration of the MRI, the ewe was positioned on its left side. The fetal femoral artery and amniotic catheters were connected to displacement transducers, a quad-bridge amplifier and a data acquisition unit (PowerLab; ADInstruments, Castle Hill, NSW, Australia) to record fetal blood pressure (corrected for amniotic pressure). All data were sampled at a rate of 1000 Hz, digitized and recorded using LabChart 7 (ADInstruments). Fetal heart rate (HR) was derived from the pressure waveform transduced from the fetal arterial catheter. The signal was processed in real-time to output an electronic trigger pulse, corresponding to each fetal heartbeat, which acted as a trigger for the MRI scanner to synchronize the image acquisition to the fetal cardiac cycle [6, 21].

Imaging was performed using a 3 Tesla clinical MRI system (MAGNETOM Skyra; Siemens Healthineers, Germany). Fetal blood flow measurements and oxygen saturations were assessed using PC MRI and T2 MRI oximetry, following previously established methods [7, 20, 21]. All maternal gas mixtures for this study were delivered through the ventilator at atmospheric pressure. MRI hemodynamic measurements were performed in three states, normoxic (maternal oxygen titrated to attain fetal conscious blood gas), hyperoxia (maternal inspiration of 100% O_2_) and hyperoxia with an infusion of phenylephrine (hyperoxia + PE; PE, 50 μg bolus and 0.1 mg/kg/min infusion; Sigma Aldrich, Castle Hill, Australia) into the fetal umbilical vein. At least 10 min elapsed between the normoxic and hyperoxic states to ensure that maternal and fetal PO_2_ and SO_2_ increased or after the beginning of the phenylephrine infusion before commencement of MRI data acquisition. Each ewe (*n* = 8) and their respective fetuses (*n* = 8) underwent all three states within a single MRI session.

### Calculation of oxygen delivery and consumption, and lower trunk flow

To determine both oxygen delivery and consumption, blood flow and T2-derived oxygen saturations were combined to calculate overall fetal and cerebral DO_2_ and VO_2_, using the following: equations 1–4. Lower trunk flow was estimated using equation 5. 


(1) \begin{align*}& \boldsymbol{Fetal}\ \boldsymbol{D}{\boldsymbol{O}}_{\mathbf{2}}\!:\nonumber\\& \quad D{O}_2=1.36\times \left[ Hb/1000\right]\times \left({Y}_{UV}/100\right)\times{Q}_{UV} \end{align*}

(2) \begin{align*}& \boldsymbol{Cerebral}\ \boldsymbol{D}{\boldsymbol{O}}_{\mathbf{2}}\!: \nonumber\\& \quad{DO}_2=1.36\times \left[ Hb/1000\right]\times \left({Y}_{AAo}/100\right)\times{Q}_{CCa} \end{align*}

(3) \begin{align*}& \boldsymbol{Fetal}\ {\boldsymbol{VO}}_{\mathbf{2}}\!:\nonumber\\ &\ \ {VO}_2=1.36\times \left[ Hb/1000\right] \times \left(\Big({Y}_{UV}-{Y}_{DAo}\right)/100\Big)\times{Q}_{UV} \end{align*}

(4) \begin{align*}& \boldsymbol{Cerebral}\ {\boldsymbol{VO}}_{\mathbf{2}}\!:\nonumber\\& \ \ {VO}_2=1.36\times \left[ Hb/1000\right] \times \left(\left({Y}_{AAo}-{Y}_{SVC}\right)/100\right)\times{Q}_{CCa} \end{align*}

(5) \begin{align*}& \boldsymbol{Lower}\ \boldsymbol{Trunk}\ \boldsymbol{flow}\!:\nonumber\\& \quad{Q}_{LT}= DAo\ \left( ml/\mathit{\min}\right)- UV\ \left( ml/\mathit{\min}\right) \end{align*}

Q_UV_ denotes the measured blood flow in the umbilical vein, while Q_CCa_ indicates the combined blood flow of the carotid arteries (CCa). [Hb] represents the mean fetal hemoglobin concentration during the MRI scan, with 1.36 representing the amount of oxygen (ml at 1 atmosphere) bound per gram of hemoglobin. Y_UV_ signifies the oxygen saturation (%) of umbilical vein blood, Y_DAo_ the oxygen saturation of descending aorta (DAo) blood, Y_AAo_ the oxygen saturation of ascending aortic blood, and Y_SVC_ the oxygen saturation of superior vena cava (SVC) blood. DO_2_ (UV and CCa) represents oxygen delivery to the fetus and the fetal brain, respectively. Q_LT_ denotes the calculated lower trunk flow.

### Postmortem and fetal weight

Following MRI studies at 123–124 d GA, pregnant ewes were humanely killed with an overdose of sodium pentobarbitone (Virbac, New South Wales, Australia). The fetus was delivered by Caesarean section, and both body and organ weights were recorded.

### Statistical analysis

Normality of the data was assessed using the Shapiro–Wilk test, and results indicated a normal distribution, with statistical significance defined as *P* < 0.05. Due to technical limitations in acquiring fetal hemodynamic measurements during the hyperoxia + PE condition, not all blood gas and fetal vessel data were obtained across all experimental states. Grubbs’ outlier test (α = 0.05) was used to identify outliers, with a significance threshold of *P* = 0.05. Fetal blood gas data were analyzed using a mixed-effects model with a Bonferroni correction for multiple comparisons. Fetal blood pressure, and flows in the UV, DV, and left ventricular cardiac output (LVCO), as well as overall fetal DO_2_ were analyzed using Repeated Measures One Way ANOVA with a Bonferroni correction for multiple comparisons. As a result of missing data points due to technical reasons, CCa, SVC, DAo, lower trunk blood flow, and fetal and cerebral VO_2_ were analyzed using mixed-effects models with a Bonferroni correction for multiple comparisons. Correlations were assessed using simple linear regression. Statistical significance was defined as *P* < 0.05.

## Results

### Fetal blood gases

Significant differences were observed in fetal blood gases across the experimental timeframe (Table 1). Fetal PaO_2_ increased significantly from normoxia to hyperoxia and then again in hyperoxia + PE. PaCO_2_ remained consistent across all states. pH values were lower during MRI compared to the morning of the MRI, while SaO_2_, Hb and Hct increased significantly from normoxia to hyperoxia + PE. There was an overall significant difference in blood lactate levels between states. However, there were no specific differences in pairwise comparisons after adjustment for multiple comparisons (Table 1).

**Table 1:** Fetal blood gases, pH, Hb, Hct, lactate prior to and during MRI data acquisition

		MRI			
	Conscious (n = 7)	Normoxia (n = 8)	Hyperoxia (n = 8)	Hyperoxia + PE (n = 8)	P-value
PaO_2_ (mmHg)	18.5 ± 2.4*^a^*	21.5 ± 2.0*^a^*	24.7 ± 2.5*^b^*	31.2 ± 5.0*^c^*	**<0.0001**
PaCO_2_ (mmHg)	50.3 ± 2.0	54.7 ± 6.7	53.6 ± 11.7	52.3 ± 8.3	0.6767
pH	7.381 ± 0.015*^a^*	7.292 ± 0.042*^b^*	7.281 ± 0.054*^b^*	7.251 ± 0.032*^b^*	**<0.0001**
SaO_2_ (%)	54.6 ± 9.8*^a^*	59.1 ± 5.8*^ab^*	66.6 ± 4.4*^ac^*	74.8 ± 8.9*^c^*	**0.0031**
Hb (g/l)	100 ± 9*^ab^*	95 ± 11*^a^*	95 ± 12*^a^*	106 ± 10*^b^*	**0.0019**
Hct (%)	30 ± 3*^ab^*	28 ± 3*^a^*	28 ± 4*^a^*	31 ± 3*^b^*	**0.0017**
Lactate (mmol/l)	1.40 ± 0.37*^a^*	1.93 ± 0.46*^a^*	1.94 ± 0.50*^a^*	2.73 ± 1.01*^a^*	**0.0082**

All fetal (*n* = 8) blood gas data were analyzed using mixed-effect analysis with a Bonferroni correction for multiple comparisons and presented as mean ± SD. *P* < 0.05 is considered statistically significant. Superscript alphabet letters (^a,b,c^) indicate a statistically significant difference between groups, with values sharing the same letter are not significantly different. One blood gas sample is missing in the conscious state due to technical issues.

### Impact of hyperoxia + PE on fetal blood pressure and heart rate

Fetal systolic blood pressure (SBP) significantly increased during both hyperoxia and hyperoxia + PE when compared to normoxia (Figure 1a). In contrast, fetal diastolic blood pressure only increased in the hyperoxia + PE state with similar levels observed between normoxia and hyperoxia (Figure 1b). Fetal mean arterial pressure (MAP) like SBP was significantly increased during both hyperoxia and hyperoxia + PE relative to normoxia (Figure 1c). While fetal HR remained unchanged during hyperoxia alone and increased only following the addition of phenylephrine (Figure 1d), indicating that elevated arterial pressure during hyperoxia was not accompanied by increased HR.

**Figure 1:**
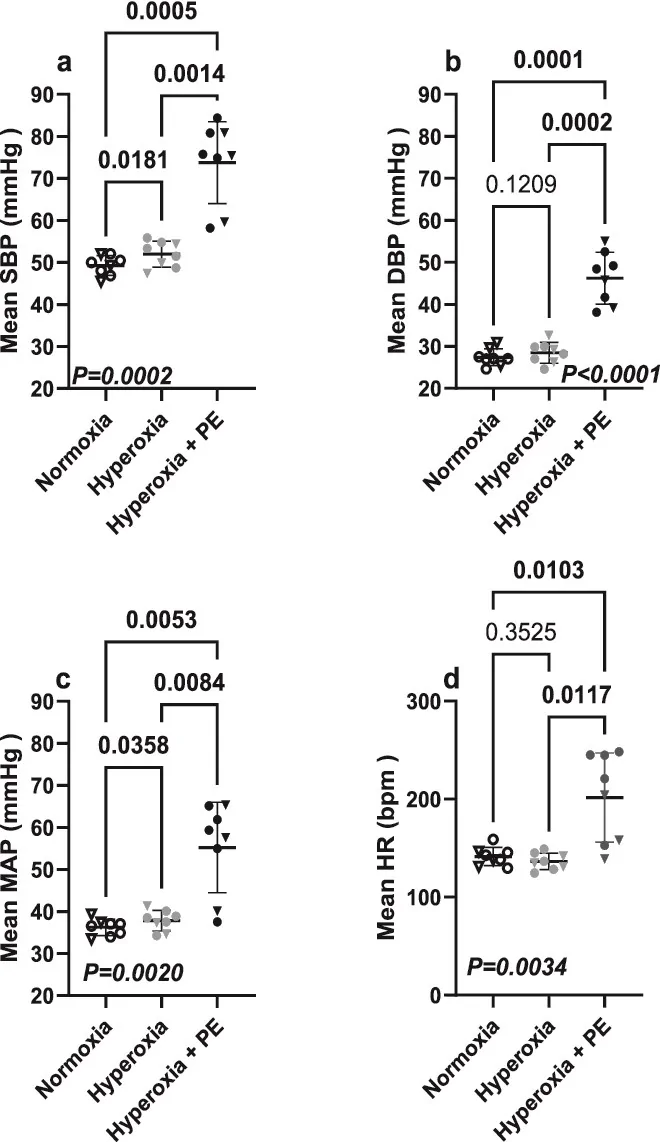
The impact of both hyperoxia and hyperoxia with the addition of fetal PE administration on fetal SBP (a), DBP (b), MAP (c) and HR (d) during the MRI session. Data is presented as individual data points with mean ± SD superimposed (*n* = 8; three males, five females) in normoxia (males ▽, females ○), maternal hyperoxia (males 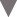, females 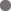) and hyperoxia + PE (males ▼, females ●). Data were analyzed by Repeated Measures One-way ANOVA with Bonferroni’s correction for multiple comparisons. *P* < 0.05 is considered statistically significant.

### Impact of hyperoxia + PE on DV flow and streaming

Fetal UV blood flow increased in hyperoxia + PE compared with hyperoxia alone (Figure 2a), accompanied by a significant increase in DV blood flow relative to both normoxia and hyperoxia (Figure 2b). Despite these increases in absolute UV and DV flow, the proportion of UV blood shunted through the DV remained unchanged across all states (Figure 2c). Simple linear regression revealed a positive association between fetal HR and both UV (Figure 2d) and DV (Figure 2e) blood flow. However, HR was not associated with relative DV shunting (Figure 2f).

**Figure 2:**
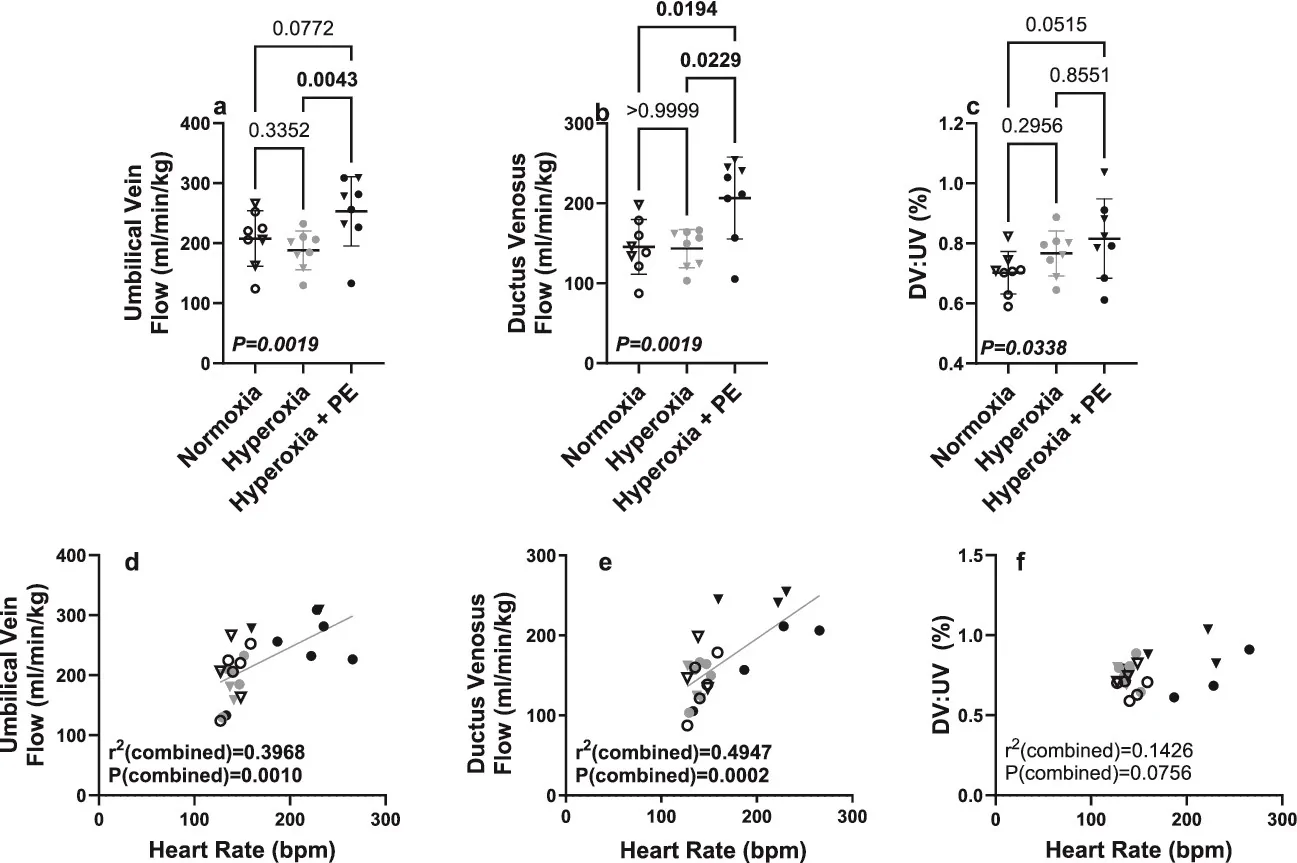
Effect of hyperoxia with or without fetal phenylephrine administration on UV (a) and DV blood flow (b) along with the overall impact on DV streaming (c). Regressions showing the relationship between fetal heart rate and UV blood flow (d), DV flow (e) and DV streaming (f) in all three states. Data is presented as individual data points with mean ± SD superimposed (*n* = 8; three males, five females) in normoxia (males ▽, females ○), maternal hyperoxia (males 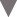, females 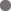) and hyperoxia + PE (males ▼, females●). Fetal blood flow data were analyzed using Repeated Measures One-way ANOVA with Bonferroni correction for multiple comparisons, and the results are presented as mean ± SD. The correlation between fetal flows and heart rate was assessed with Simple Linear Regression. Both analyses considered *P* < 0.05 as statistically significant.

### Effect of hyperoxia + PE on fetal hemodynamics

Hyperoxia + PE significantly increased LVCO compared with both normoxia and hyperoxia alone (Figure 3a). In contrast, blood flow in the fCCAs was significantly reduced in the hyperoxia + PE state (Figure 3b). Reductions in blood flow were also observed in the DAo (Figure 3c), SVC (Figure 3,d), and lower trunk (Figure 3e). Despite these hemodynamic effects, the proportion of blood flow between the fCCA and lower trunk did not change between states (Figure 3f).

**Figure 3:**
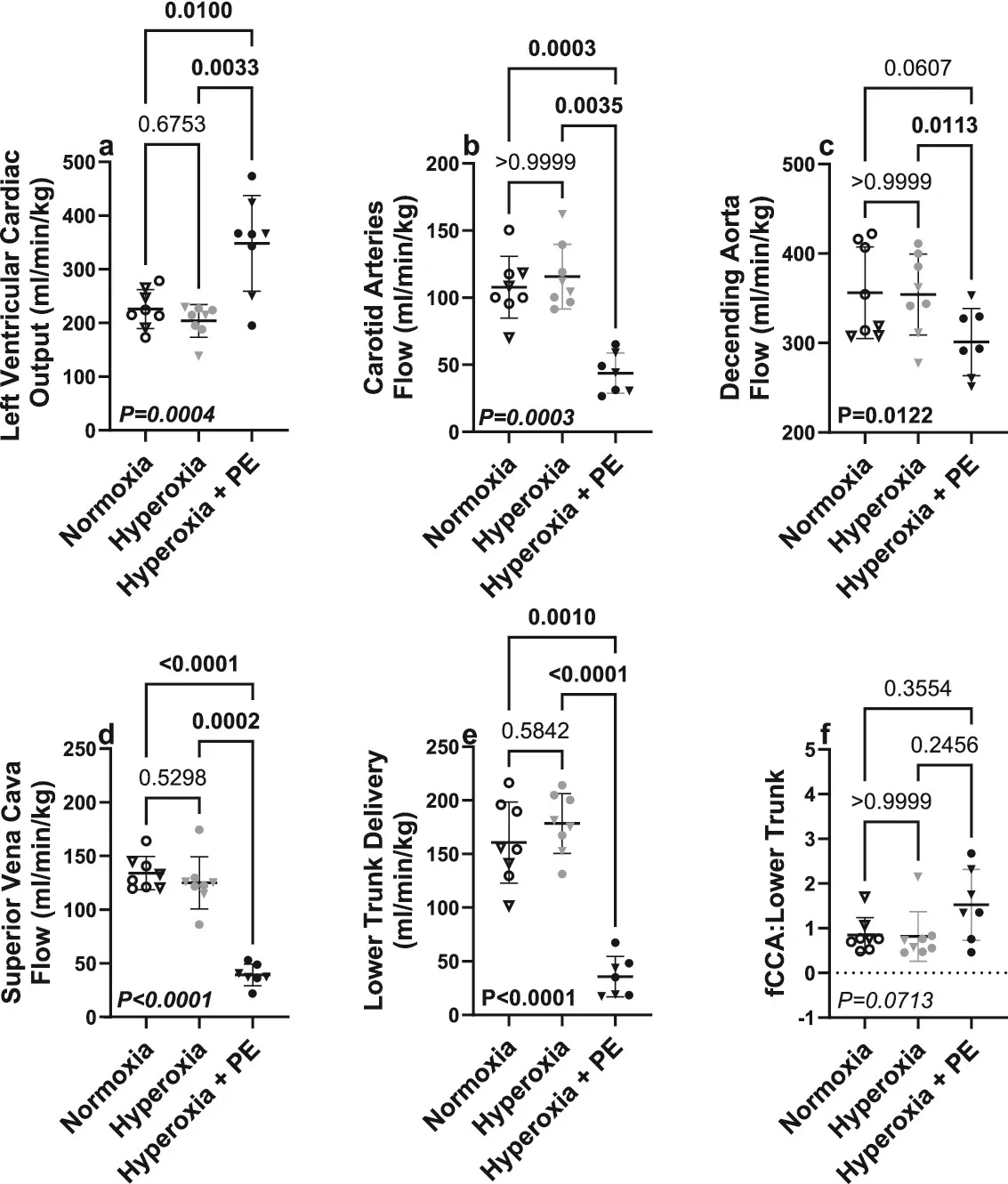
LVCO (a), combined carotid arteries (b), DAo (c), SVC (d), lower trunk flow (e) and combined carotid arteries to lower trunk ratio (f) in normoxia, hyperoxia and hyperoxia + PE. Data presented as individual data points in normoxia (males ▽, females ○), maternal hyperoxia (males 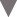, females 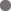) and hyperoxia + PE (males ▼, females ●). Due to technical limitations during hyperoxia + PE, complete vessel acquisition was not achieved for one fetus, resulting in a reduced sample size (n = 7; three males, four females). All data were analyzed using a mixed-effects analysis with Bonferroni correction for multiple comparisons and are reported as mean ± SD with *P* < 0.05 considered statistically significant.

### Effect of hyperoxia and PE on oxygen delivery and consumption

Fetal DO_2_ was significantly increased in response to hyperoxia + PE (Figure 4a), while Feal VO_2_ remained unchanged between states (Figure 4b). Despite the increase in overall fetal DO_2_, cerebral DO_2_ decreased during hyperoxia + PE (Figure 4c) with no change in cerebral VO_2_ (Figure 4d). A reduction in the proportion of fetal DO_2_ contributing to cerebral DO_2_ was observed in hyperoxia + PE (Figure 4e).

**Figure 4:**
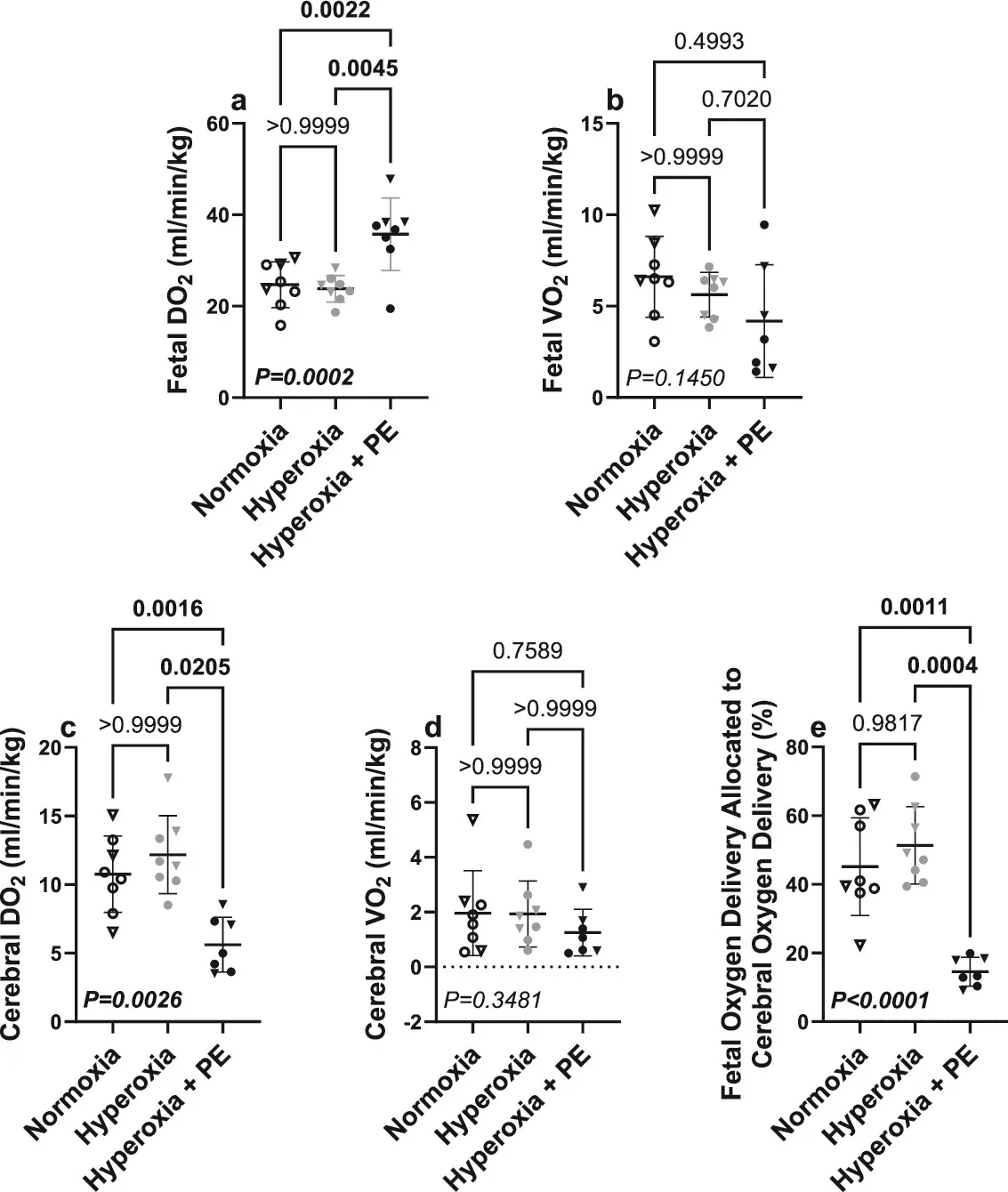
Comparison of fetal DO_2_ (a) and VO_2_ (b) and cerebral DO_2_ (c) and VO_2_ (d) calculated using previously described PC MRI derived blood flow measurements, oxygen saturation using T2 oximetry and fetal blood hemoglobin concentrations in MRI. Fetal DO_2_ allocated to cerebral DO_2_ (e) was calculated by dividing cerebral DO_2_ / fetal DO_2_ ×100. Fetal DO_2_ data is presented as individual data points with mean ± SD superimposed (*n* = 8; three males, five females) in normoxia (males ▽, females ○), hyperoxia (males 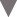, females 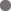) and hyperoxia + PE (males ▼, females ●). Data were analyzed using a Repeated Measures one-way ANOVA with Bonferroni’s correction for multiple comparisons and presented as mean ± SD. *P* < 0.05 is considered statistically significant. Due to technical limitations during hyperoxia + PE, complete vessel acquisition was not achieved for one fetus, resulting in a reduced sample size (*n* = 7; three males, four females) for the fetal VO_2_ (b), cerebral DO_2_ (c) cerebral VO_2_ (d) and the percentage of fetal DO_2_ that becomes cerebral DO_2_ (e). This data was analyzed using a mixed-effects analysis with Bonferroni correction for multiple comparisons and are reported as mean ± SD with *P* < 0.05 considered statistically significant.

## Discussion

Acute perturbations of fetal oxygenation, specifically during acute hypoxemia, alter the diameter of the DV through α-adrenergic mediated hepatic vascular vasoconstriction, preferentially increasing DO_2_ to vital fetal organs such as the heart and brain [9, 13]. This observation is due to increased blood flow returning from the placenta via the UV travelling through the DV instead of perfusing the liver [13]. Although the effects of fetal hypoxemia on the DV are well characterized, the impact of fetal hyperoxia remains unclear. Given that maternal hyperoxygenation has been proposed as a potential intervention to improve outcomes in compromised fetuses [34–36], it is essential to explore the possible impact this may have on fetal hemodynamics; in particular, hemodynamics known to be sensitive to changes in fetal DO_2_.

The premise behind maternal hyperoxygenation as a therapy is that by increasing maternal oxygenation, fetal DO_2_ will also increase and thus resolve the fetal hypoxemia. However, components of the fetal vasculature are sensitive to changes in oxygen availability, causing hyperoxia itself to alter fetal hemodynamics [34, 36, 37]. This includes changes in DV diameter, which reduces as fetal hypoxemia subsides and the elevated catecholamine signaling is no longer required [18, 34, 37–41] as well as increases to pulmonary blood flow through oxygenation induced dilation of the pulmonary vasculature [34]. Although theoretically beneficial overall, fetal hyperoxia necessitates further mechanistic investigation to understand its impact on specific pathways. In the present study, an α-adrenergic receptor agonist, was used to pharmacologically induce hepatic vasoconstriction, with the goal of increasing DV shunting despite prevailing fetal hyperoxia. In this study, α-adrenergic agonism paired with fetal hyperoxia significantly increased absolute UV and DV flow compared to both the normoxia and hyperoxia states. Despite this increased DV flow, there were no changes in the proportion of UV blood directed through the DV (DV:UV %; DV shunting). This is indicative of the α-adrenergic agonist exerting systemic effects rather than its effecting being only isolated to the hepatic vasculature. Given this systemic impact and the presence of α-adrenergic receptors in the developing myocardium [42, 43], the α-adrenergic agonist also significantly increased fetal heart rate. This increase in heart rate is likely the driver of the observed increase in LVCO. Notably, α-adrenergic agonism increased blood pressure, likely via α-mediated vasoconstriction.

Continued investigation of downstream vascular responses revealed that α-adrenergic agonism significantly reduced carotid blood flow, a decrease not observed in the hyperoxia only state. The literature surrounding the impact of α-adrenergic stimulation of the carotid arteries is limited and has varied findings. However, our data is in line with ex *vivo* wire myography studies reporting carotid artery constriction in response to α-adrenergic agonism, indicating carotid adrenergic innervation [44, 45]. In addition, cerebral DO₂ was reduced in the hyperoxia + PE state without parallel changes in cerebral VO₂. This reduction is consistent with the accompanying decrease in combined carotid blood flow, indicating reduced oxygen delivery to the fetal brain. Collectively, this data emphasizes that increased oxygen and blood flow via the DV do not guarantee increased cerebral DO_2_ when α-adrenergic tone is elevated. When hyperoxia was combined with α-adrenergic agonism, LVCO increased alongside a reduction in combined carotid flow, indicating that elevated downstream cerebrovascular resistance can attenuate the impact of increased upstream DV and UV flow. Another potential explanation for this result is that the reduction in cerebral blood flow may represent a protective response by the fetal brain to prevent hyperoxia induced injury through the generation of reactive oxygen species (ROS) [46]. Studies in rats show that hyperoxic conditions increase ROS production and subsequent vascular dysfunction [47]. However, the proposed mechanism for this protective response warrants further investigation to fully elucidate its implications and underlying pathways.

Previous work has demonstrated that isoflurane produces a dose-dependent cerebral vasodilation and blunted autoregulation, therefore, flow becomes more passive to changes in blood pressure [48]. The introduction of an α-adrenergic agonist may further complicate this state. Although PE is commonly used to counteract isoflurane induced hypotension, its impact on cerebral oxygenation remains inconsistent. Multiple human studies, including those conducted under anesthesia, demonstrate that while PE reliably increases blood pressure, it simultaneously reduces cerebral oxygenation [49, 50]. This suggests that increased blood pressure does not necessarily translate to improved cerebral perfusion. Which is in line with the presence of protective cerebral autoregulatory mechanisms and in the case of the present study, anesthesia induced vasodilation, vasopressor induced flow shifts, and oxygen driven flow through the DV [51]. The fetal sheep cerebral vessels can be stimulated by α-adrenergic agonist when applied directly [52]. However, PE does not readily cross the blood brain barrier due to its hydrophilic nature, thus making a direct impact on the central nervous system an unlikely contributor to the findings in this study [53, 54]. Cerebral blood is regulated automatically across a range of blood pressures [55] and thus, the reduction in carotid artery blood flow and cerebral DO_2_ and the concurrent increase in MAP observed in the hyperoxia + PE state is most likely attributed to the systemic peripheral impacts of PE, rather than a direct effect of PE on the fetal brain’s vasculature.

Importantly, the fetuses in this study were normoxic and from uncomplicated pregnancies, and thus baseline oxygenation was not impaired. In comparison to the vasodilatory and cardiovascular changes triggered by hypoxemia or pharmacological challenges, in both preterm and term neonates as well as in fetal sheep studies have demonstrated that a transient increase in fetal oxygenation can lead to cerebral vasocontraction [56–58] a neuroregulatory response may be activated to protect the fetal brain from excessive oxygen exposure once a specific threshold is exceeded. This interpretation is supported by molecular evidence demonstrating that the brain is highly sensitive to oxidative stress, with hyperoxia induced ROS production linked to mitochondrial dysfunction and neuronal damage [59]. This is further supported by the observation that, despite the reduction in cerebral DO_2_, oxygen consumption remained unchanged, indicating that the brain did not require more oxygen in this context.

This study has several limitations that should be acknowledged. PE was administered to pharmacologically impact α-adrenergic tone with specific focus on the liver. However, its effects were not confined to the hepatic vasculature, as evidenced by systemic changes in HR, arterial pressure, and thus cardiac output. Consequently, the observed increases in DV, UV and AAo flow likely reflect broader cardiovascular effects rather than being due to selective modulation of DV tone. In addition, all MRI data collection was performed under general anesthesia, which can alter fetal hemodynamics, including vascular tone, pressure and flow. Isoflurane induces vasodilation and blunting of autoregulatory mechanisms may therefore have influenced baseline hemodynamics and modified the fetal response to both hyperoxia and α-adrenergic stimulation. A further limitation of this study is the lack of pulmonary blood flow measurements, especially given the pulmonary vasculature is sensitivity to oxygen mediated vasodilation [60]. Future studies should specifically examine how these interventions affect pulmonary vascular responses. Finally, due to time constraints in the scanner, we were unable to investigate hemodynamic and oxygenation changes in a normoxia+PE state. However, although the normoxia+PE state was not feasible within the present study, the fetal response to PE has been well characterized in chronically instrumented conscious fetal sheep across gestation [30, 61–63]. PE increased fetal MAP in both preterm and near-term fetuses, providing the expected direction and magnitude of blood pressure change in response to PE in the fetus with a normoxic state [61–63]. Although the hemodynamic response reported in this study is consistent with previous work describing the α-adrenergic agonism in the fetus [64, 65], they should be interpreted in the context of the developmental regulation of the α-adrenergic system whereby both innervation density, receptor expression, and response magnitude increase with advancing gestational age [66]. Therefore, functional α-adrenergic responses are present at the gestational age studied, although their magnitude may be less early in gestation or greater near or at term [64–66]. All these factors should be considered when interpreting the findings of this study.

To the best of our knowledge, this study is the first to comprehensively examine the effects of maternal hyperoxygenation, both alone and in combination with α-adrenergic agonism on the fetal circulation, using clinically relevant MRI techniques. We demonstrate that hyperoxygenation alone does not alter DV flow or shunting (DV: UV flow ratio) to the brain. In contrast, the addition of PE during hyperoxia increases UV, DV, and AAo blood flow, reflecting enhanced cardiac output. However, these upstream changes did not alter cerebral DO_2_. Instead, α-adrenergic agonism during hyperoxia is associated with reduced carotid blood flow and the consequent reduction in cerebral DO₂, despite increased overall fetal DO_2_. These findings indicate that increased DV flow under hyperoxic+PE condition does not necessarily enhance cerebral oxygenation when downstream cerebrovascular resistance is elevated and suggest that α-adrenergic influences on the circulation of the carotid arteries may play an important role in regulating fetal cerebral perfusion. Future studies should aim to disentangle the independent effects of hyperoxia and α-adrenergic stimulation, including the addition of a normoxia + PE state, and explore alternative strategies to selectively modulate hepatic or DV tone without eliciting unintended systemic or cerebrovascular effects.

## Data Availability

The data that support the findings are included in the manuscript and any MRI images can be made available from the corresponding author upon reasonable request.

## Additional information

No additional information.
